# Bone grafting in pedicle screw fixation of upper cervical spine: is it necessary?

**DOI:** 10.1097/JS9.0000000000002399

**Published:** 2025-04-10

**Authors:** Guiguan Wang, Jie Xu

**Affiliations:** a Shengli Clinical Medical College of Fujian Medical University, Fuzhou, Fujian, China; b Department of Orthopedic, Fujian Provincial Hospital, Fuzhou, Fujian, China; c Fujian Provincial Clinical Medical Research Center for Spinal Nerve and Joint Diseases, Fuzhou, Fujian, China;; d Fuzhou University Affiliated Provincial Hospital, Fuzhou, Fujian, China

## Introduction

The atlantoaxial complex is essential for maintaining cervical spine stability and enabling rotational movement, making it a critical focus in upper cervical spine surgery. Pedicle screw fixation is a widely accepted technique for managing instability in this region^[^1^]^. Traditionally, bone grafting on the posterior arch is an essential part of this procedure to enhance fusion and stability, although chronic pain at the bone graft donor site is common^[^2^]^. However, no studies have reported on the outcomes of pedicle screw fixation without bone grafting. This retrospective case series, with at least 10 years follow-up, aims to investigate the possibility of avoiding bone grafting in pedicle screw fixation by comparing the fusion rates at the posterior arch grafting site with the rates of spontaneous fusion at the lateral masses.

## Methods

All patients with atlantoaxial instability or dislocation who underwent pedicle screw fixation and posterior arch fusion using iliac bone graft at a single hospital between January 2012 and December 2014, with complete medical records, were retrospectively included. This study was approved by the independent ethics committee.

Perioperative management was standardized for all patients. The pedicle screw fixation technique followed Tan’s method^[^3^]^. Three-dimensional cervical CT scans were used to evaluate the fixation and bone fusion. Bone fusion was defined as continuous trabecular bone bridging between the posterior arch and iliac bone graft, or continuous trabecular bone bridging across the atlantoaxial joint with indistinct joint margins.

Continuous data were expressed as mean ± standard deviation and categorical variables were represented as data (percentage). Differences were compared using the chi-square test, with *P* < 0.05 considered statistically significant.

**HIGHLIGHTS**

- 82.76% of patients achieved spontaneous fusion at the lateral mass joints despite grafting being performed on the posterior arch.
- The high rate of lateral mass fusion contrasts with the notable resorption observed in posterior arch grafting, suggesting that grafting in the posterior arch may provide limited long-term benefits.

## Results

**Figure 1.**
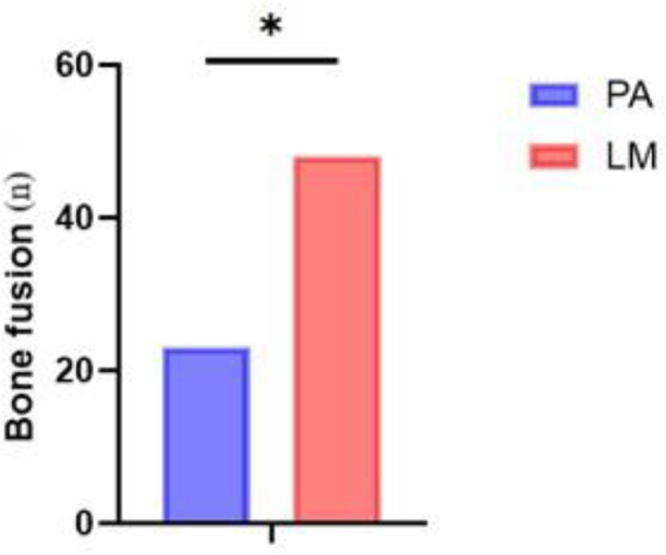
The number of cases with bone fusion occurring at only one site. Abbreviations: PA, posterior arch; LM, lateral mass. *P < 0.001.

Of 78 patients screened, 11 were lost to follow-up, and 7 deceased patients reported no cervical discomfort (Table 1). Sixty completed follow-up. Two patients using 3.5 mm screws experienced breakage: one with ankylosing spondylitis achieved fusion despite breakage; the other required reoperation with 4.0 mm screws and additional bone grafting after initial fusion failure. Among the 58 patients with over 10 years of follow-up, no cervical discomfort was reported. Bone fusion occurred in 10 cases at the posterior arch graft site, 13 at both the lateral mass joints and posterior arch, and 35 at the lateral mass joints with posterior graft resorption (Figure 1).

**Table 1:** Perioperative characteristics

Preoperative characteristics	
Number of cases	58/78 (74.36%)
Age (years)	39.9 ± 16.1
Female (%)	28 (48.28%)
Height (m)	1.62 ± 0.12
Weight (kg)	65.50 ± 11.80
Follow-up time	128.92 ± 7.85
Bone fusion rates	
PA	23 (39.66%)
LM	48 (82.76%)
Both PA and LM	13 (22.41%)

Abbreviations: PA: posterior arch; LM: lateral mass.

## Discussion

This study provides critical evidence that spontaneous fusion in the lateral mass joints is feasible and may obviate the necessity for routine bone grafting in this region. Our findings demonstrated that 82.76% of patients achieved spontaneous fusion at the lateral mass joints despite grafting being performed solely on the posterior arch. This underscores the significance of biomechanical forces, as described by Wolff’s law, in driving bone remodeling and fusion at horizontal weight-bearing surfaces^[^4^]^.

Intraoperative lateral mass reduction and firm internal fixation likely created an optimal environment for spontaneous fusion by stabilizing the joint and enabling sustained physiological loading. The high rate of lateral mass fusion contrasts with the notable resorption observed in posterior arch grafting, suggesting that grafting in the posterior arch may provide limited long-term benefits. These findings challenge the conventional reliance on posterior arch grafting and emphasize the potential for simplifying surgical techniques without compromising outcomes.

Our study supports the possible application of individualized approaches to atlantoaxial stabilization, emphasizing fixation strategies that leverage natural bone-healing mechanisms. Future research should focus on prospective trials to validate these findings and explore alternative fixation techniques that maximize fusion rates while minimizing surgical complexity and donor site morbidity.

## Data Availability

The original contributions presented in the study are included in the article/supplementary material. Further inquiries can be directed to the corresponding authors.

## Research registration unique identifying number (UIN)

The registration number is researchregistry11150.
